# Oral Chinese Herbal Medicine as an Adjuvant Treatment for Chemotherapy, or Radiotherapy, Induced Myelosuppression: A Systematic Review and Meta-Analysis of Randomized Controlled Trials

**DOI:** 10.1155/2017/3432750

**Published:** 2017-08-10

**Authors:** Bonan Hou, Rui Liu, Zhen Qin, Dan Luo, Qi Wang, Shuiqing Huang

**Affiliations:** ^1^School of Basic Medicine, Guangzhou University of Chinese Medicine, Guangzhou, Guangdong, China; ^2^Department of Pharmacology, Guiyang Medical University, Guiyang, Guizhou, China; ^3^Institute of Clinical Pharmacology, Guangzhou University of Chinese Medicine, Guangzhou, Guangdong, China

## Abstract

**Objective:**

Myelosuppression is a common side effect in cancer patients receiving chemotherapy or radiotherapy. Chinese herbal medicine (CHM) has shown promise in alleviating myelosuppression.

**Method:**

We searched for randomized controlled trials (RCTs) from seven databases without language restriction. We included RCTs in adults, in which hematological toxicity was measured according to WHO criteria and control group underwent chemotherapy and/or radiotherapy and the treatment group was given oral CHM.

**Results:**

We searched 1021 articles from the date of databases inception to October 7, 2016. We selected 14 articles for the final analysis. Pooled data showed that CHM significantly decreased the suppression rate of leukocytes, neutrophils, hemoglobin, and platelets compared with the control group, particularly in grade III-IV toxicity (leukocytes: RR = 0.43, 95% CI = 0.33–0.56; neutrophils: RR = 0.39, 95% CI = 0.27–0.58; hemoglobin: RR = 0.33, 95% CI = 0.18–0.61; platelets: RR = 0.61, 95% CI = 0.39–0.95).

**Conclusions:**

CHM as an adjuvant can alleviate myelosuppression induced by chemotherapy or radiotherapy, reduce grade III-IV toxicity, and maintain therapeutic dose and treatment cycle. However, due to heterogeneity and publication bias, the results should be interpreted with caution and validated by conducting strictly designed multicenter RCTs of high quality and large scale.

## 1. Introduction

Myelosuppression, also known as bone marrow (BM) suppression or myelotoxicity, is the major side effect of chemotherapy and radiotherapy. Blood originates in the BM [1] and blood cells have limited life [2]. Once myelosuppression is induced, BM is unable to maintain normal levels of blood cells, which results in a series of complications, such as anemia, infection, and tendency of hemorrhage. Moreover, chemotherapy induces dose-limiting myelosuppression toxicity, but dose reductions would affect treatment efficacy. Therefore, myelosuppression should be addressed while considering chemotherapy or radiotherapy.

It is vital to improve survival rate of cancer patients being treated for anemia, infection, and other complications. However, some treatments could cause adverse effects. For example, G-CSF causes shock or chronic fibrous pneumonia and repetitive platelet transfusion leads to formation of alloantibody [3]. Therefore, it is crucial to find safe agents that can reduce myelosuppression and improve survival rate of patients receiving chemotherapy or radiotherapy. In recent years, Chinese herbal medicine (CHM) has shown promise in this regard.

Studies on CHM preventing myelosuppression have been inconclusive or conflicting. Currently, it is difficult to make the clinical decision of administering CHM as an adjuvant in chemotherapy or radiotherapy. A similar systematic review was published [4], which revealed effects of various CHMs in myelosuppression prevention. To provide better insights for making appropriate clinical decisions, we aimed to review the effects of oral CHM as we believe that the effects vary with the route of administration.

In this study, our objective was to minimize clinical heterogeneity and pool high-quality studies to generate robust evidence regarding the potential therapeutic value of CHM in preventing myelosuppression. This meta-analysis was reported and performed in accordance with PRISMA guidelines (S1 File in Supplementary Material available online at https://doi.org/10.1155/2017/3432750).

## 2. Methods

### 2.1. Inclusion and Exclusion Criteria

We included studies that conducted randomized controlled trials (RCTs) and in which patients were over 18 years of age, control group was treated with radiotherapy or drug therapy and treatment group was treated with CHM along with radiotherapy or drug therapy, and hematological toxicity was measured mainly according to World Health Organization (WHO) criteria [19] (Table 1). The exclusion criteria were as follows: (1) assessment of hematological toxicity by total bone marrow inhibition rate, rather than WHO criteria; (2) treatment with acupuncture, intravenous CHM, CHM granules, patented CHM drug, or CHM extract; (3) treating patients in control group with CHM and not chemotherapy or radiotherapy; (4) treating patients in treatment group with more than two types of CHM; and (5) methodological quality score of less than 3 points on Jadad scale.

### 2.2. Search Strategy

We systematically searched databases, including China Academic Journal Network Publishing Database (CAJD), China Biology Medicine disc (CBMdisc), China Doctoral Dissertations Full-text Database (CDFD), China Master's Dissertations Full-text Database (CMFD), PubMed, Cochrane, and EMBASE, for relevant articles published from the date of the databases inception to October 7, 2016. There were no language restrictions. As part of search strategy, we used a combination of the following terms: “Chinese herbal medicine” (“Chinese medicine,” “traditional Chinese medicine (TCM),” “Chinese herbal medicine,” “Chinese herbal drug,” “traditional herbal medicine,” “herbal medicine,” “traditional Japanese medicine,” “traditional medicine,” “ethnomedicine,” “folk medicine,” “folk remedies,” “indigenous medicine,” “primitive medicine,” “materia medica,” “homeopathic remedies,” “traditional East Asian medicine,” “traditional Far Eastern medicine,” “Far East medicine,” “Oriental medicine,” “Korean medicine,” “Tibetan medicine,” “herb,” “herbaceous agent,” “medicinal plant,” “medicinal herbs,” “medicinal plant product,” “plant preparation,” “herbal preparation,” “botanic,” “botany” “Kampo,” “traditional Mongolian medicine,” “Mongolian folk medicine,” “Mongolian medicine,” “phytotherapy,” “herb therapy,” “herbal therapy,” “ethnopharmacology,” “alternative medicine,” “alternative therapy,” “complementary therapy,” “complementary medicine,” “TCM,” “CHM,” “tang,” or “decoction”) [20] and “radiotherapy” (“drug therapy” or “chemotherapy”) and “randomized controlled trial” (“randomized controlled trial,” “controlled clinical trial,” “randomized controlled trials,” “random allocation,” or “random^*∗*^”).

Gray articles were identified by searching Chinese Clinical Trial Register (ChiCTR), the American Society of Clinical Oncology (ASCO), and Google. In addition, we manually searched reference lists of relevant articles.

### 2.3. Study Selection and Data Extraction

Studies were selected by two researchers (BNH, RL). First, titles were read and irrelevant or duplicate articles were removed. Second, editorials, opinions, or case reports were excluded through abstract screening. Third, after reading the full text, on the basis of inclusion and exclusion criteria, articles were filtered. BNH and RL selected articles independently and any disagreement was resolved by discussion. If they still disagreed, the third reviewer (SQH) intervened to resolve the disagreement.

Data were extracted by two researchers (DL, ZQ) separately, including authors, years, cancer type, number of patients (including number of dropouts or withdrawals), intervention, outcome, detailed content on CHM, and Jadad score. The extracted data were verified by the third author (QW). Disagreements were resolved by face-to-face discussion among the three authors.

### 2.4. Quality Assessment

Methodological qualities of the included RCT trials were assessed using Jadad scale [21]. Two researchers (BNH, RL) assessed all articles and disagreements were resolved by discussion with a third researcher (SQH).

### 2.5. Statistical Analysis

The statistical analysis was performed using Stata 14.0. For dichotomous outcomes, relative risk (RR) and 95% confidence interval (CI) were used. The model of random effects or fixed effects was applied according to heterogeneity of pooled data. Heterogeneity was assessed by Cochran's* Q* and the *I*^2^ statistics [22]. In random effects model, *I*^2^ of ≥50% or *P* value of ≤0.10 indicated significant heterogeneity, whereas, in fixed effects model, *I*^2^ of ≤50% and *P* value of ≥0.10 indicated homogeneity. If heterogeneity was significant, we performed sensitivity analysis to identify the possible sources.

When meta-analysis included no less than 10 articles, Begg, Egger, and Harbord tests were used to evaluate the publication bias [23–25].

## 3. Results

### 3.1. Article Selection

**Figure 1:**
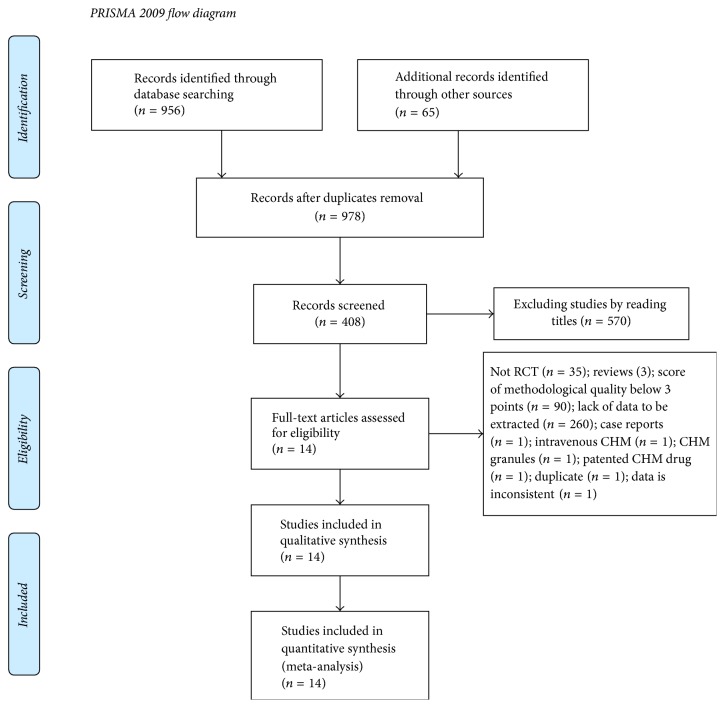
Flow chart of article search.

We retrieved 1021 articles through databases and Internet search as well as manual search. After removing duplicates, 978 articles were identified, and, after reading titles, 570 irrelevant articles were excluded and 408 potentially relevant articles were included. Following abstract screening, 3 reviews and 1 case report were excluded. Finally, through a conscientious review of full text, 390 articles were removed, including studies of low methodological quality (90 articles); studies with duplicate (1 article), data inconsistency (1 article), or no RCT (35 articles); studies that lack sufficient data (260 articles); and studies that used intravenous CHM (1 article), CHM granules (1 article), or patented CHM drug (1 article). Thus, 14 articles [5–18] were included.  Figure 1 presents a flow chart of the study selection process.

### 3.2. Characteristics of Articles Included

In total, 1275 patients (14 articles) were considered, 65 dropped out or withdrew [5–7, 9, 11–18], 71 lacked myelosuppression data [8], and 62 took CHM at different time points [18]. Finally, 1077 patients were included.  Table 2 shows the characteristics of the articles included. Cancers included ovarian cancer (3 articles), breast cancer (3 articles), throat cancer (1 article), non-small-cell lung cancer (6 articles), and colorectal cancer (1 article). All articles were graded at least 3 on Jadad scale and one was graded 5. CHM was administered to patients twice a day during chemotherapy and radiotherapy.

### 3.3. Myelosuppression

#### 3.3.1. Effects of CHM on WBC

**Figure 2:**
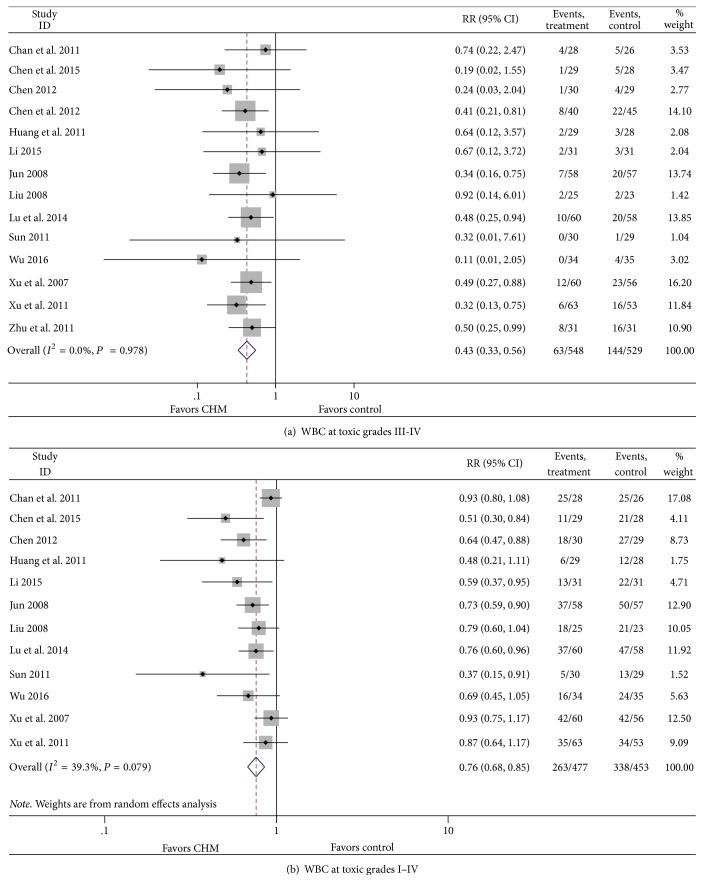
Effect of CHM on leukocytes.

The number of patients in CHM group at toxic grades III-IV and I–IV in whom WBC inhibition was observed was less than that in the control group (RR = 0.43, 95% CI = 0.33–0.56, *P* < 0.001; RR = 0.74, 95% CI = 0.67–0.81, *P* < 0.001), with no heterogeneity in grades III-IV (*I*^2^ = 0%, *P* = 0.978). However, in grades I–IV, there was significant heterogeneity among the articles (*I*^2^ = 49.5%, *P* = 0.026). For searching possible sources of heterogeneity, we excluded articles one by one. After dropping “Chan 2011,” heterogeneity was *I*^2^ = 20.3%, *P* = 0.250 (random effects model) or *I*^2^ = 24.1%, *P* = 0.214 (fixed effects model) (Figure 2).

#### 3.3.2. Effect of CHM on Neutrophils

**Figure 3:**
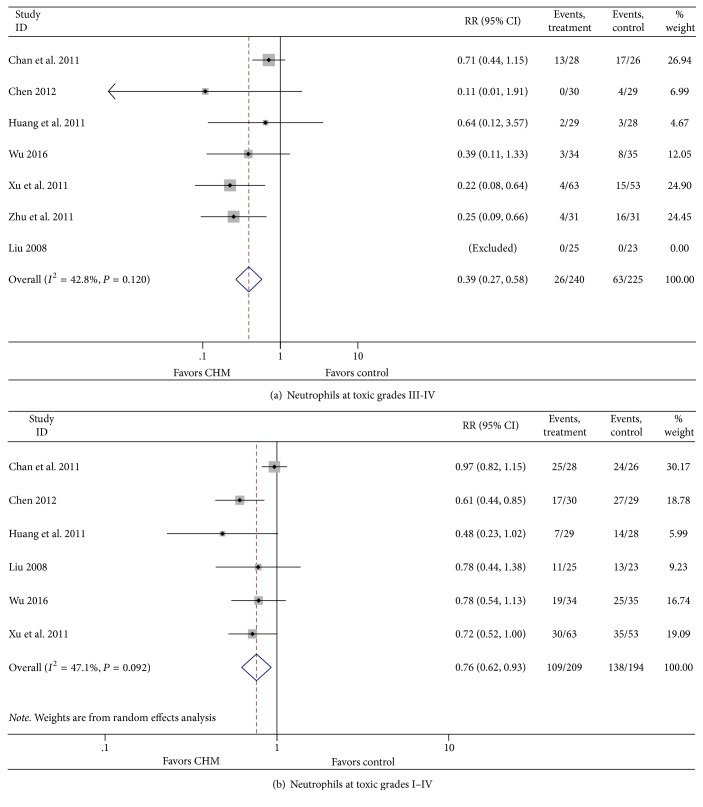
Effect of CHM on neutrophils.

The suppression rate of neutrophils inpatients belonging to the CHM group at toxic grades III-IV was only 39% of that in the control group (RR = 0.39, 95% CI = 0.27–0.58, *P* < 0.001), and, at toxic grades I–IV, the rate was 76% of that in the control group (RR = 0.76, 95% CI = 0.62–0.93, *P* = 0.008). There was no heterogeneity in toxic grades III-IV (*I*^2^ = 42.8%, *P* = 0.120); however, in grades I–IV, significant heterogeneity was observed among articles (*I*^2^ = 47.1%, *P* = 0.092). For searching possible sources of heterogeneity, we excluded articles one by one. After dropping “Chan 2011,” heterogeneity was *I*^2^ = 0.0%, *P* = 0.709 (random effects model) or *I*^2^ = 0.0%, *P* = 0.709 (fixed effects model) (Figure 3).

#### 3.3.3. Effect of CHM on HB

**Figure 4:**
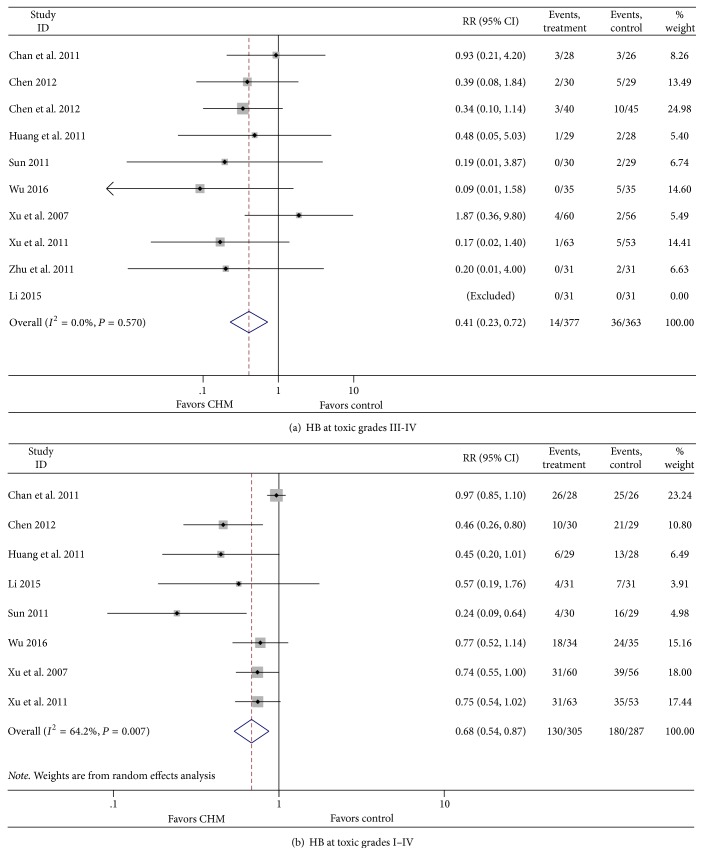
Effect of CHM on hemoglobin.

In the CHM group, the suppression of HB decreased by 67% in patients at toxic grades III-IV and 30% in patients at toxic grades I–IV (RR = 0.41, 95% CI = 0.23–0.72, *P* = 0.002; RR = 0.68, 95% CI = 0.54–0.87, *P* = 0.002), compared with the control group, with no heterogeneity in grades III-IV (*I*^2^ = 0%, *P* = 0.570) but with significant heterogeneity in grades I–IV (*I*^2^ = 64.2%, *P* = 0.007). On further exclusion of articles one by one, we found that dropping of “Chan 2011” decreased heterogeneity [*I*^2^ = 28.9%, *P* = 0.208 (random effects model) and *I*^2^ = 33.9%, *P* = 0.169 (fixed effects model)] (Figure 4).

#### 3.3.4. Effect of CHM on PLT

**Figure 5:**
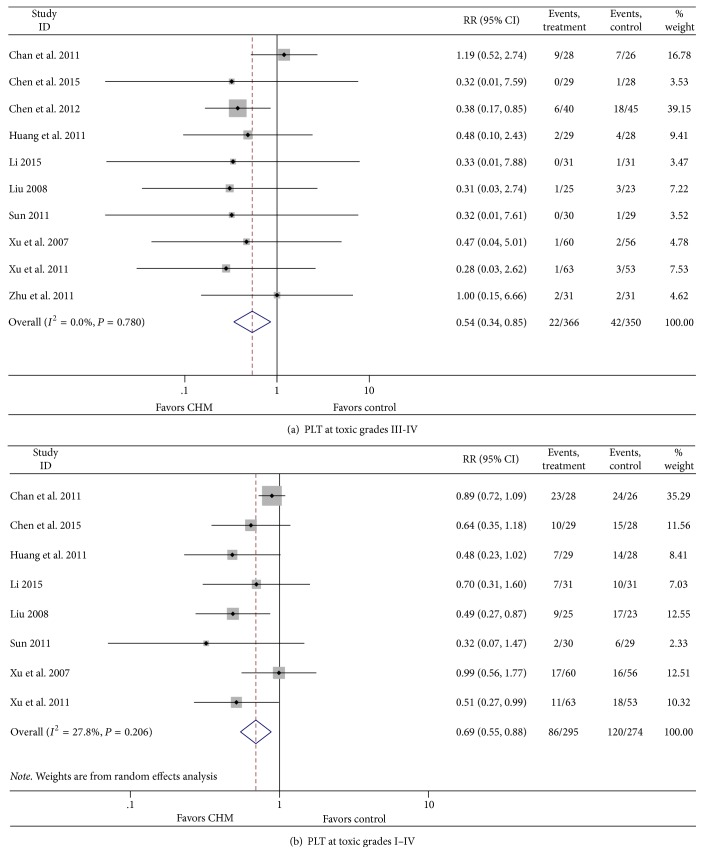
Effect of CHM on platelets.

In the CHM group, the number of patients at toxic grades III-IV and I–IV in whom PLT inhibition was observed was less than that in the control group (RR = 0.54, 95% CI = 0.34–0.85, *P* = 0.028; RR = 0.69, 95% CI = 0.55–0.88, *P* = 0.003), with no heterogeneity in grades III-IV (*I*^2^ = 0%, *P* = 0.780) and I–IV (*I*^2^ = 27.8%, *P* = 0.206) (Figure 5).

#### 3.3.5. Subgroup Analysis of WBC at Toxic Grades II–IV

**Figure 6:**
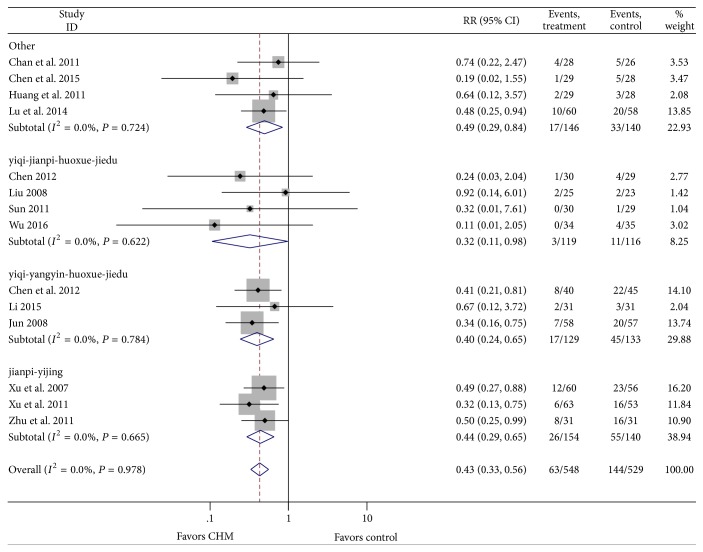
Subgroup analysis of WBC at toxic grades III-IV.

According to the theory for TCM, we divided 14 articles by the treatment into 4 subgroups: yiqi-jianpi-huoxue-jiedu, yiqi-yangyin-huoxue-jiedu, jianpi-yijing, and other. Result indicated that all 4 types of treatments were effective (RR = 0.322, 95% CI = 0.106–0.979, *P* = 0.046; RR = 0.397, 95% CI = 0.242–0.650, *P* < 0.001; RR = 0.438, 95% CI = 0.294–0.654, *P* < 0.001; RR = 0.494, 95% CI = 0.291–0.839, *P* = 0.009), with no heterogeneity (Figure 6).

### 3.4. Publication Bias and Sensitivity Analysis

There were 4 meta-analyses including no less than 10 articles; of them, one showed the presence of a publication bias (Table 3). The remaining articles had no evidence to indicate the presence of potential publication bias.

We analyzed sensitivity by changing the analysis model (Table 4). The RR of pooling data was robust.

## 4. Discussion

We performed meta-analysis of CHM as an adjuvant treatment for chemotherapy- or radiotherapy-induced myelosuppression. The results suggested that oral CHM is effective in protecting WBC, neutrophils, HB, and PLT. Begg, Egger, and Harbord tests revealed the presence of publication bias in WBC (toxic grades I–IV) analysis. The sensitivity analysis indicated that all analyses were robust with no evident publication bias.

In this meta-analysis, the curative effect for grades III-IV was better than that for I–IV. The myelosuppression rate at toxic grades I-II showed no significant difference among the CHM treatment, no-CHM treatment, and placebo treatment groups (supplementary document). Result suggested that toxic grades I-II were responsible for the difference between toxic grades III-IV and toxic grades I–IV. We further analyzed the results for toxic grades I-II. In general, toxic grades III-IV were the index for the clinical treatment of patients showing myelosuppression, in whom treatment for toxic grade II or lower mainly focuses on symptomatic therapy. These results were similar to those in published articles about treating myelosuppression inpatients mostly at toxic grades III-IV. Source data from the 14 articles was insufficient to confirm our hypothesis about toxic grades I-II. We did not analyze toxic grades I, II, III, IV, and II–IV, as they provided no extra information.

The main finding of our meta-analysis was different from that of the previous one [4], which indicated that CHM significantly protected peripheral WBC, but not peripheral HB or PLT. We found that CHM can protect not only peripheral WBC, but also HB, PLT, and neutrophils. The previous study analyzed continuous data from 8 articles but did not pool data for neutrophils. Our meta-analysis pooled ranked data for WBC, neutrophils, HB, and PLT from 14 articles. Moreover, the referenced articles were different. We did not include the articles in the previous meta-analysis, as seven of eight articles did not meet our inclusion criteria and the remaining one was of low methodological quality (Jadad scale < 3 score). In addition, different data style may induce different results. Ranked data reflects the curative effect of treating various patients under serious conditions, rather than a simple summary that would conceal effective treatment, such as continuous data.

To obtain robust results in the present meta-analysis, we included high-quality RCTs using Jadad scale to screen the methodological quality of articles and excluded studies that treated patients with acupuncture, intravenous CHM, CHM granules, patented CHM drug, or CHM extract, to reduce clinical heterogeneity among articles for comparison with previous meta-analyses. According to the theory for TCM, the herbs used in the 14 articles belong to yiqi-jiedu (tonifying qi and disintoxication) treatment. Our meta-analysis only included oral CHM; the results showed low heterogeneity (WBC, HB, and neutrophils at toxic grades I–IV) and homogeneity, which suggested that the yiqi-jiedu treatment could be the same treatment. To validate our hypothesis, we performed subgroup analysis of WBC at toxic grades III-IV (only WBC at toxic grades III-IV was included in all 14 articles). The 14 articles were divided by the herb into 4 subgroups, which means that yiqi-jiedu treatment was also subdivided into 4 treatments. The results were similar. Moreover, sensitivity analysis based on change of analysis model did not fundamentally alter most of the pooled results.

In Asia, CHM has long been used in the treatment of chronic diseases and cancer as a primary therapy or adjuvant therapy. Nevertheless, one may assume that CHM played the role of placebo. Two of the 14 articles reported that the control group received placebo [5, 13], which was indistinguishable in terms of taste and appearance from the study medication. There was no significant placebo effect. Furthermore, several high-quality articles suggested that CHM was not a potential placebo [26, 27].

Some articles indicated that there was a positive interaction between the administered CHM and concomitant chemotherapy or radiotherapy [28, 29]. The administration of CHM along with chemotherapy may offer some immediate benefits to patients such as improvement of tumor response and quality of life. However, in myelosuppression, there was a negative interaction between the two therapies. The therapeutic mechanism of CHM as an adjuvant treatment for myelosuppression remains unclear. The dominant opinion about the effectiveness of CHM is that it alleviates myelosuppression by multitarget treatments [30, 31].

Neutropenia is a common dose-limiting toxicity that is difficult to treat. Our meta-analysis indicated that oral CHM as an adjuvant can prevent the decrease in neutrophils during chemotherapy and radiotherapy, suggesting that CHM maintains the therapeutic dose and treatment cycle. Furthermore, CHM can prevent the decrease in WBC, HB, and PLT. Compared with the other treatments, the advantage of CHM is that it evidently improves the curative effect of chemotherapy and radiotherapy.

Our meta-analysis has some limitations. First, CHM treatment is an individualized therapy. According to the theory for TCM, the difference in individualized therapy should be based on different diseases or symptoms. In our meta-analysis, the treatments in 14 articles belonged to yiqi-jiedu. Furthermore, our results show low heterogeneity and homogeneity and the results of subgroup analysis are similar. Although clinical heterogeneity is low, division into 4 subgroups could improve the robustness of the results. However, as the present research is limited, it was difficult to perform further analysis. Second, our meta-analysis did not include results for lymphocytes. Neutrophils: lymphocyte ratio is also an important hematological toxicity predictive index. However, there are no relevant articles meeting our inclusion criteria. Third, we included 5 types of cancers, with different drug sensitivity. Fourth, only two articles mention the concrete blind method. Fifth, we could not assess the impact of CHM treatment on other clinically meaningful endpoints, such as overall survival and quality of life, due to the limited number of reports available.

Future studies could focus on the following points. First, drugs for tonifying qi and disintoxication play an important role in the treatment. Therefore, these types of drugs should be studied, as further subdividing treatment could provide more robust results, new therapy ideas, or drugs against myelosuppression. Second, better blind methods should be designed to improve methodological quality. Third, further studies can focus on other clinical endpoints, such as quality of life, survival, and length of stay, as there is a lack of high-quality studies demonstrating the correlation between CHM and these endpoints.

## 5. Conclusions

Evidence obtained from this study suggests that CHM can be used as an adjuvant to alleviate myelosuppression induced by chemotherapy or radiotherapy and, in particular, reduce grade III-IV toxicity. However, due to heterogeneity and publication bias in the partial results of this meta-analysis (WBC at toxic grades I–IV, neutrophils at toxic grades I–IV), the results should be interpreted with caution and validated by conducting strictly designed multicenter RCTs of high quality and large scale.

## Supplementary Material

S1: PRISMA guidelines.

## Figures and Tables

**Table 1:** Recommendation for grading of WHO criteria.

	Grade 0	Grade I	Grade II	Grade III	Grade IV
Hemoglobin (HB)	≥11.0 g/100 ml	9.5–10.9 g/100 ml	8.0–9.4 g/100 ml	6.5–7.9 g/100 ml	<6.5 g/100 ml
	≥110 g/l	95–109 g/l	80–94 g/l	65–79 g/l	<65 g/l
	≥6.8 mmol/l	5.6–6.7 mmol/l	4.95–5.8 mmol/l	4.0–4.9 mmol/l	<4.0 mmol/l

Leukocytes (WBC) (1000/mm^3^)	≥4.0	3.0–3.9	2.0–2.9	1.0–1.9	<1.0

Granulocytes (1000/mm^3^)	≥2.0	1.5–1.9	1.0–1.4	0.5–0.9	<0.5

Platelets (PLT) (1000/mm^3^)	>100	75–99	50–74	25–49	<25

**Table 2:** Characteristics of the articles included.

Authors	Years	Kind of cancer	Number of patients/dropout or withdrawal	Intervention (C/T)^a^	Outcome	Detailed content of CHM	Jadad score
Chan et al. [5]	2011	Ovarian cancers	81/27	Chemotherapy and placebo/chemotherapy and CHM	QLQ-C30^b^; the side effects of chemotherapy (the WHO criteria); immune function	BASIC FORMULA	5
Chen et al. [6]	2015	Breast cancer	60/3	Chemotherapy/chemotherapy and CHM	The side effects of chemotherapy; KPS score; immune function; serum tumor markers	Shuganjianpi decoction	3
Chen [7]	2012	Ovarian cancers	60/1	Chemotherapy/chemotherapy and CHM	The side effects of chemotherapy; KPS score; TCM syndrome index; QOL^c^	Yiliu decoction	3
Chen et al. [8]	2012	Throat cancer	156/0	Chemoradiotherapy/chemoradiotherapy and CHM	The side effects of chemotherapy (the WHO criteria); KPS score; survival time	Qingliulianghou decoction	3
Huang et al. [9]	2011	Non-small-cell lung cancer	60/3	Chemotherapy/chemotherapy and CHM	The side effects of chemotherapy (the WHO criteria); TCM syndrome index; KPS score	Yiqiyangyin decoction	3
Li [10]	2015	Colorectal cancer	62/0	Chemotherapy/chemotherapy and CHM	The side effects of chemotherapy (the WHO criteria)	Yiqiyangxue decoction	3
Jun [11]	2008	Non-small-cell lung cancer	129/14	Chemoradiotherapy/chemoradiotherapy and CHM	The side effects of chemotherapy (the WHO criteria); KPS score	Fuzhengkangai decoction	3
Liu [12]	2008	Ovarian cancers	50/2	Chemotherapy/chemotherapy and CHM	KPS score; QOL; the side effects of chemotherapy	Fuzhengquyu decoction	3
Lu et al. [13]	2014	Breast cancer	120/2	Chemotherapy and placebo/chemotherapy and CHM	The side effects of chemotherapy; use of GCSF^d^; safety evaluation	Wenshen Shengbai decoction	3
Sun [14]	2011	Non-small-cell lung cancer	60/1	Chemotherapy/chemotherapy and CHM	UICC; the side effects of chemotherapy (the WHO criteria); KPS score	Fuzhengjiedu decoction	3
Wu [15]	2016	Breast cancer	70/1	Chemotherapy/chemotherapy and CHM	The side effects of chemotherapy (the WHO criteria); immune function	Fuzhengxiaoyan decoction	3
Xu et al. [16]	2007	Non-small-cell lung cancer	120/4	Chemotherapy/chemotherapy and CHM	UICC; the side effects of chemotherapy (the WHO criteria); KPS score; survival time; weight	Kangliuzengxiao decoction and feiyanning decoction	3
Xu et al. [17]	2011	Non-small-cell lung cancer	120/4	Chemotherapy/chemotherapy and CHM	QOL; the side effects of chemotherapy (the WHO criteria); survival time	Kangliuzengxiao decoction and feiyanning decoction	3
Zhu et al. [18]	2011	Non-small-cell lung cancer	127/3	Chemotherapy/chemotherapy and CHM	TCM syndrome index; KPS score; immune function; the side effects of chemotherapy	Kangliuzengxiao decoction	3

^a^Control group/treatment group.

^b^Quality of Life Questionnaire C30.

^c^Quality of life.

^d^Granulocyte cell stimulating factor.

**Table 3:** Result of publication bias.

	Begg	Egger	Harbord
WBC III-IV^a^	*P* = 0.511	*P* = 0.553	*P* = 0.488
WBC I–IV^b^	*P* = 0.011	*P* = 0.001	*P* = 0.001
HB III-IV	*P* = 0.348	*P* = 0.166	*P* = 0.364
PLT III-IV	*P* = 0.721	*P* = 0.469	*P* = 0.384

^a^III-IV = at toxic grades III-IV; ^b^I–IV = at toxic grades I–IV.

**Table 4:** Sensitivity analysis.

Analysis	Sensitivity analysis	Heterogeneity		Pooled RR (95% CI)	
		*I* ^2^	Cochran Q	RR (95% CI)	*P*
WBC III-IV^a^	FM	0%	*P* = 0.978	0.43 (0.33–0.56)	*P* < 0.001
	RM	0%	*P* = 0.979	0.44 (0.34–0.58)	*P* < 0.001
WBC I–IV^b^	FM	49.5%	*P* = 0.026	0.74 (0.67–0.81)	*P* < 0.001
	RM	39.3%	*P* = 0.079	0.76 (0.68–0.85)	*P* < 0.001
Neutrophils III-IV	FM	42.8%	*P* = 0.120	0.39 (0.27–0.58)	*P* < 0.001
	RM	33.6%	*P* = 0.184	0.40 (0.23–0.69)	*P* = 0.001
Neutrophils I–IV	FM	60.8%	*P* = 0.026	0.73 (0.63–0.85)	*P* < 0.001
	RM	47.1%	*P* = 0.092	0.76 (0.62–0.93)	*P* = 0.007
HB III-IV	FM	0%	*P* = 0.570	0.41 (0.23–0.72)	*P* = 0.002
	RM	0%	*P* = 0.582	0.45 (0.24–0.83)	*P* = 0.002
HB I–IV	FM	81.9%	*P* < 0.0001	0.67 (0.58–0.78)	*P* = 0.011
	RM	64.2%	*P* = 0.007	0.68 (0.54–0.87)	*P* = 0.002
PLT III-IV	FM	0%	*P* = 0.780	0.54 (0.34–0.85)	*P* = 0.008
	RM	0%	*P* = 0.789	0.58 (0.36–0.93)	*P* = 0.023
PLT I–IV	FM	45.2%	*P* = 0.078	0.67 (0.54–0.82)	*P* < 0.001
	RM	27.8%	*P* = 0.206	0.69 (0.55–0.88)	*P* = 0.003

^a^III-IV = at toxic grades III-IV; ^b^I–IV = at toxic grades I–IV; FM = fixed effects model; RM = random effects model.
